# Supplementary material for: Metabolic reprogramming from glycolysis to fatty acid uptake and beta-oxidation in platinum-resistant cancer cells
Source: Nat Commun. 2022 Aug 5;13:4554. doi: 10.1038/s41467-022-32101-w (PMC9356138; doi:10.1038/s41467-022-32101-w)
Supplement: Supplementary file 1 — Supplementary Information [file 41467_2022_32101_MOESM1_ESM.pdf]

SUPPLEMENTARY INFORMATION

Metabolic Reprogramming from Glycolysis to Fatty Acid Uptake and beta-Oxidation in Platinum-Resistant Cancer Cells

Yuying Tan, Junjie Li, Guangyuan Zhao, Kai-Chih Huang, Horacio Cardenas, Yinu Wang, Daniela Matei, Ji-Xin Cheng

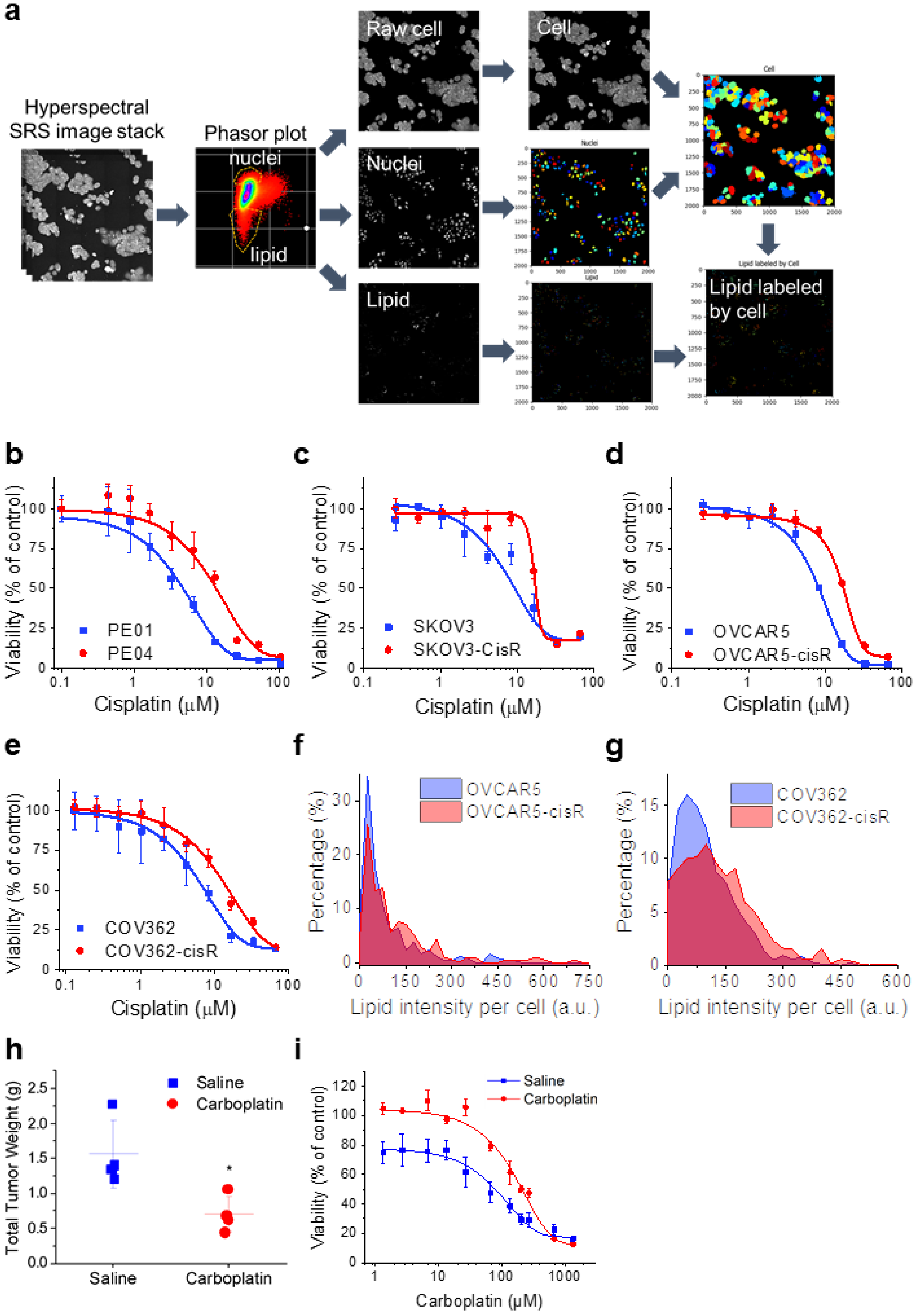
a
Hyperspectral
SRS image stack
Phasor plot
nuclei
lipid
Raw cell
Nuclei
Lipid
Cell
Lipid labeled
by cell
b
PE01
PE04
Viability (% of control)
Cisplatin (μM)
c
SKOV3
SKOV3-CisR
d
OVCAR5
OVCAR5-cisR
e
COV362
COV362-cisR
f
OVCAR5
OVCAR5-cisR
Percentage (%)
Lipid intensity per cell (a.u.)
g
COV362
COV362-cisR
h
Saline
Carboplatin
Total Tumor Weight (g)
i
Saline
Carboplatin
Viability (% of control)
Carboplatin (μM)

Supplementary Fig. 1. High-throughput profiling of lipid metabolism in ovarian cancer cell lines. (a) Image processing flow for high-throughput single cell analysis of lipids. A hyperspectral SRS image stack was segmented through spectral phasor analysis to generate segmented images of nuclei and lipid, which together with raw cell image were input in CellProfiler to outline each individual cells. Lipids were color-coded based on the colors of their parental cells. Quantitative analysis of cell morphology, lipid quantity and intensity were also produced along with the images. The image area is 500 µm by 500 µm. **(b-e)** Dose-response to cisplatin in PEO1 & 4 (b), SKOV3 & -cisR (c), OVCAR5 & -cisR (d), COV362 & -cisR cells (e). n = 6 biological replicates. **(f-g)** Histograms of integrated cellular lipid intensity in OVCAR5 & -cisR (f) and COV362 & -cisR cells (g). **(h)** Weights of xenografts from mice treated with saline or carboplatin for 3 weeks. (n=4 mice, two-sided Student's t test; P=0.030; * $P < 0.05$). **(i)** Dose-response to carboplatin in OC cells derived from xenografts developed in mice treated with carboplatin or saline. The dose-response curves and scatter plot are shown as means ± SD, n=4 technical replicates. Source data are provided in Source Data file.

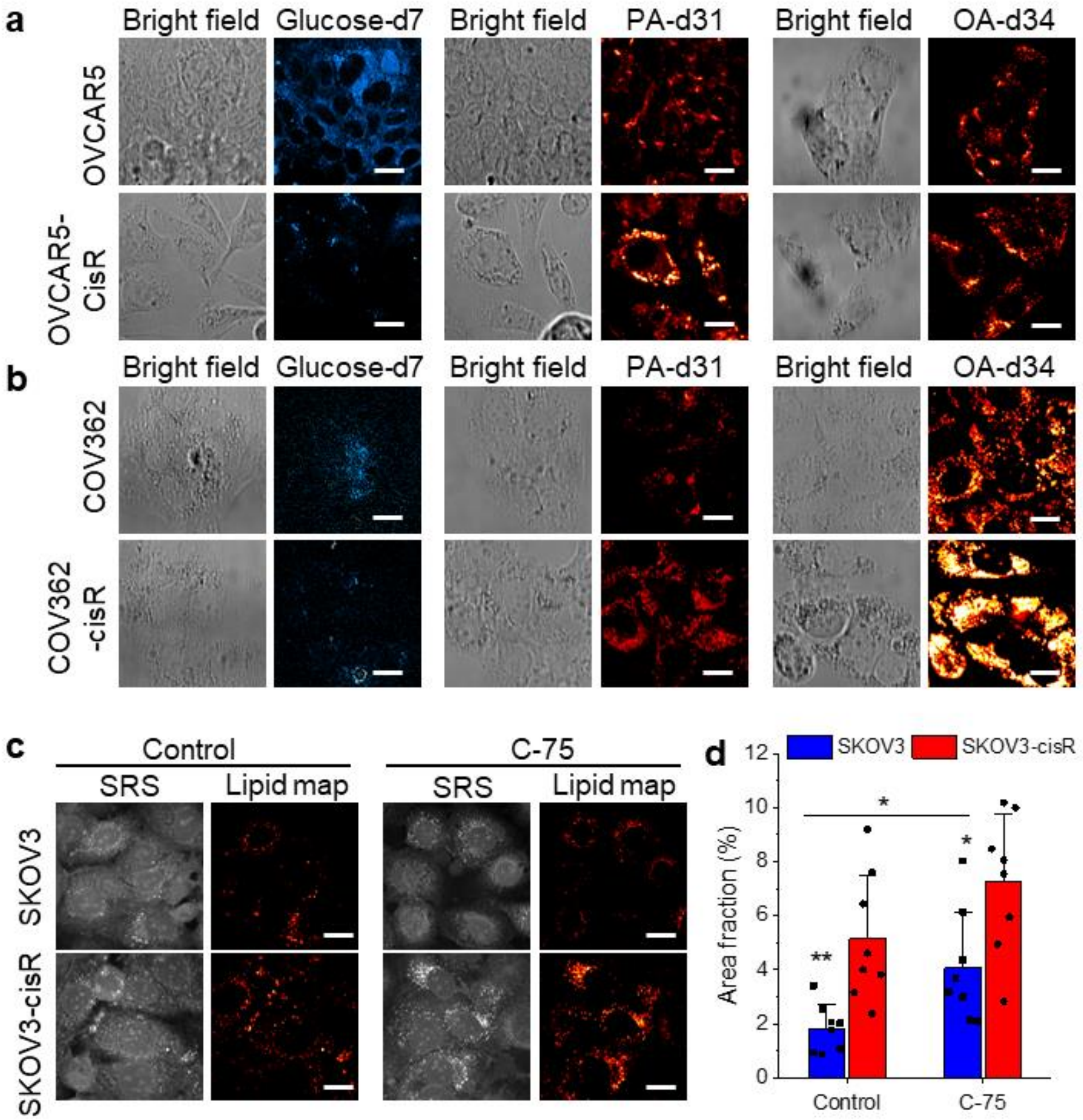

Supplementary Fig. 2. Increased FA uptake, not de novo lipogenesis, is the major contributor to lipid accumulation in cisplatin-resistant ovarian cancer cells. (a) Representative bright field and SRS images of OVCAR5 and OVCAR5-cisR cells fed with glucose-d_7 for 3 days, PA-d_{31} for 6 h, or OA-d_{34} for 6 h. **(b)** Representative bright field and SRS images of COV362 and COV362-cisR cells fed with glucose-d_7 for 3 days, PA-d_{31} for 6 h, or OA-d_{34} for 6 h. Quantification result for (a) and (b) are shown in Supplementary Table 1. n=6 technical replicates. **(c)** Representative sum of hSRS and phasor mapped lipid image of SKOV3 and -cisR cells treated with vehicle or 10 μM C-75. **(d)** Quantitative analysis of SRS signal from lipid in SKOV3 and -cisR cells treated with vehicle or 10 μM C-75. The results are shown as means + SD; n = 8 technical

replicates; two-sided Student’s t test; P=0.0050, 0.019 and 0.016; * $P < 0.05$ and ** $P < 0.01$. All scale bar: 20 µm. Source data are provided in Source Data file.

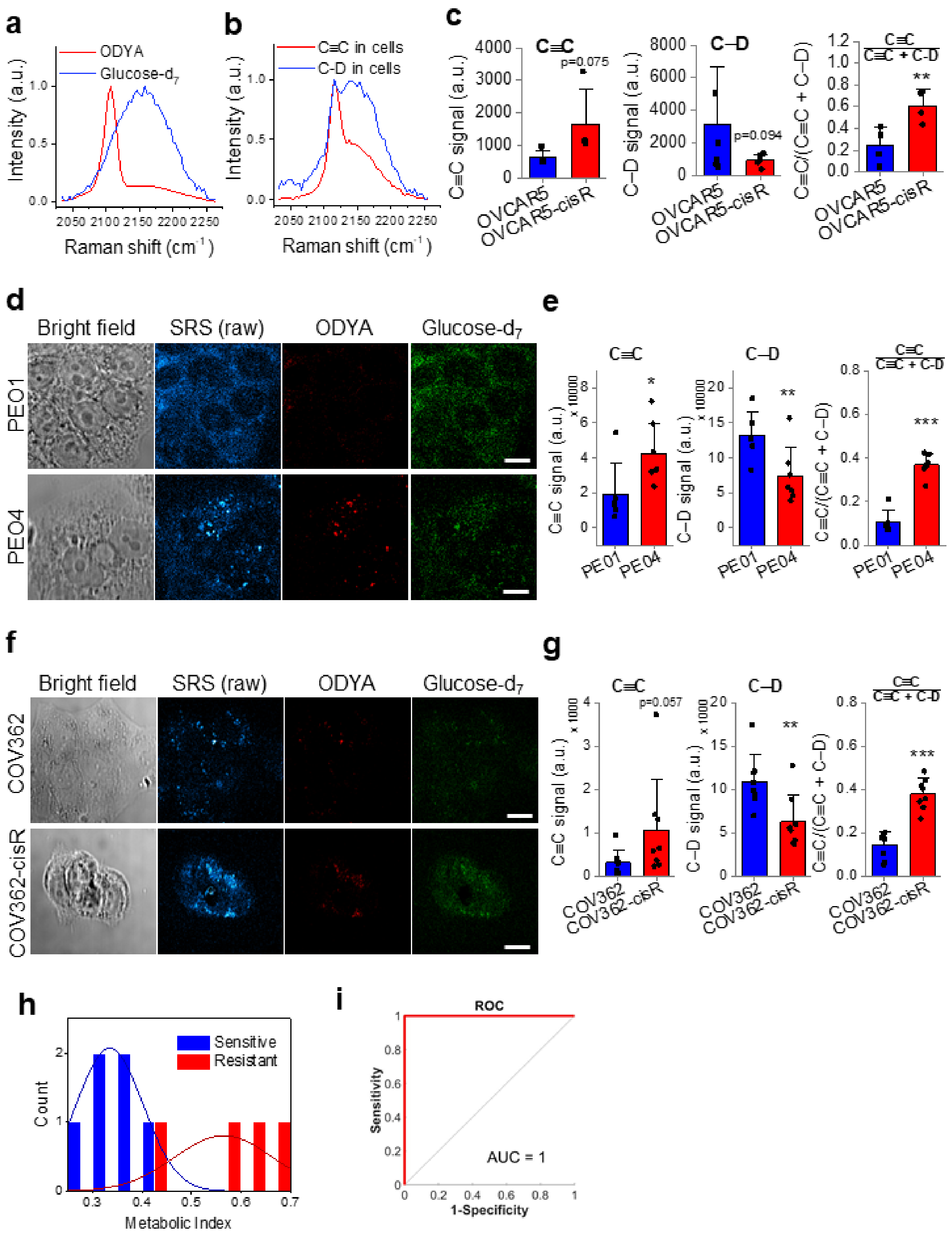
a
ODYA
Glucose-d7
Intensity (a.u.)
Raman shift (cm-1)
b
C≡C in cells
C-D in cells
Intensity (a.u.)
Raman shift (cm-1)
c
C≡C
C–D
p=0.075
p=0.094
C≡C signal (a.u.)
C–D signal (a.u.)
C≡C/(C≡C + C–D)
OVCAR5
OVCAR5-cisR
d
Bright field
SRS (raw)
ODYA
Glucose-d7
PEO1
PEO4
e
C≡C signal (a.u.)
C–D signal (a.u.)
PE01
PE04
f
COV362
COV362-cisR
g
p=0.057
h
Sensitive
Resistant
Count
Metabolic Index
i
ROC
Sensitivity
1-Specificity
AUC = 1

Supplementary Fig. 3. Metabolic index calculated by integrating glucose derived lipogenesis and FA uptake directly correlates with cisplatin resistance. (a) Normalized SRS spectra of ODYA and glucose-d7 in cells. **(b)** Output SRS spectra from phasor analysis of C≡C bonds from ODYA and C-D bonds from glucose-d_7 and metabolites. **(c)** Quantitative analysis of ODYA derived C≡C intensity (n=4), glucose-d_7 derived C-D intensity (n=6), and the ratio of C≡C/(C≡C + C-D) (n=4) in OVCAR5 and OVCAR5-cisR cells. P=0.0082. **(d)** Representative bright field images, raw SRS images, and processed SRS images of ODYA and glucose-d_7 in PEO1 and PEO4 cells. **(e)** Quantitative analysis of ODYA derived C≡C intensity, glucose-d_7 derived C-D intensity, and the ratio of C≡C/(C≡C + C-D) in PEO1 (n=6) and PEO4 (n=7) cells. P=0.018, 0.0093 and 1.5×10^{-6}. **(f)** Representative bright field images, raw SRS images, and processed SRS images of ODYA and glucose-d_7 in COV362 and COV362-cisR cells. Scale bar: 20 µm. **(g)** Quantitative analysis of ODYA derived C≡C intensity, glucose-d_7 derived C-D intensity, and the ratio of C≡C/(C≡C + C-D) in COV362 and COV362-cisR cells (n=8). P=0.057, 0.0047 and 2.9×10^{-6}. n for (b-g) represents technical replicates. Data in all the bar charts (c, e, and g) are shown as means + SD. All scale bar: 20 µm. All statistical significance was analyzed using one-sided Student's t test. * $P < 0.05$, ** $P < 0.01$, and *** $P < 0.001$. **(h)** Histograms of metabolic index of primary ovarian cancer cells from platinum resistant patients (n=6 biological replicates) and platinum sensitive patients (n=4 biological replicates). **(i)** ROC curve for metabolic index of primary ovarian cancer cells from patients with platinum resistant or platinum sensitive tumors. AUC: area under curve. Source data are provided in Source Data file.

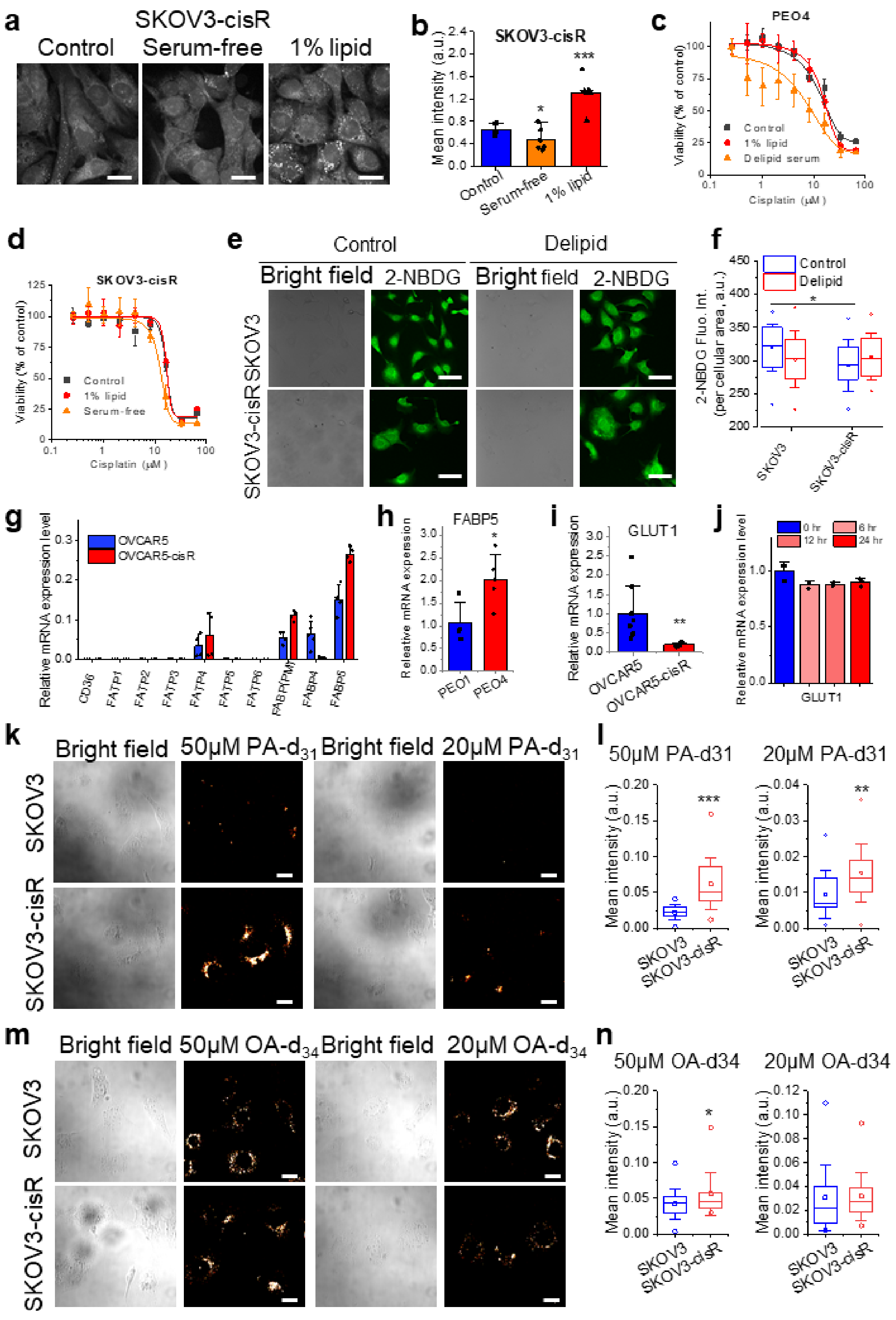
a
SKOV3-cisR
Control
Serum-free
1% lipid
b
SKOV3-cisR
Mean intensity (a.u.)
Control
Serum-free
1% lipid
c
PEO4
Viability (% of control)
Control
1% lipid
Delipid serum
Cisplatin (μM)
d
SKOV3-cisR
Viability (% of control)
Control
1% lipid
Serum-free
Cisplatin (μM)
e
Control
Delipid
Bright field
2-NBDG
SKOV3
SKOV3-cisR
f
Control
Delipid
2-NBDG Fluo. Int. (per cellular area, a.u.)
SKOV3
SKOV3-cisR
g
OVCAR5
OVCAR5-cisR
Relative mRNA expression level
CD36
FATP1
FATP2
FATP3
FATP4
FATP5
FATP6
FABP4
FABP5
h
FABP5
Relative mRNA expression
PEO1
PEO4
i
GLUT1
Relative mRNA expression
OVCAR5
OVCAR5-cisR
j
0 hr
6 hr
12 hr
24 hr
Relative mRNA expression level
GLUT1
k
Bright field
50μM PA-d31
20μM PA-d31
SKOV3
SKOV3-cisR
l
50μM PA-d31
20μM PA-d31
Mean intensity (a.u.)
SKOV3
SKOV3-cisR
m
Bright field
50μM OA-d34
20μM OA-d34
SKOV3
SKOV3-cisR
n
50μM OA-d34
20μM OA-d34
Mean intensity (a.u.)
SKOV3
SKOV3-cisR

Supplementary Fig. 4. FA uptake directly contributes to cisplatin resistance. (a-b) Representative SRS images and C-H signal from lipid droplet quantitation of SKOV3-cisR cultured with control serum (FBS) (n=5), no serum (n=6) and control serum supplemented with 1% lipid mixture (n=6) for 24 hours. Scale bar: 20 µm. P=0.044 and 0.00089. n represents technical replicates. **(c-d)** Dose-response to cisplatin with control, reduced (delipid or no serum) and increased (control serum with 1% lipid mixture) lipid content media for PEO1 (c), SKOV3 (d). n = 3 biological replicates. **(e-f)** Representative bright field and fluorescent images, and fluorescent signal quantification of SKOV3 (n=19 and 20) and -cisR (n=16) treated with 100 µM 2-NBDG for 2 hours after cultured in control or reduced lipid content medium (delipid) for 24 hours. Scale bar: 50 µm. P= 0.024. n represents technical replicates. **(g)** Relative mRNA expression of CD36, FATP1-6, FABP4-5, and FABP PM in OVCAR5 and -cisR. n = 2 for FATP6; n=4 for FABP4 and FATP4; n=3 for other genes. **(h)** Relative mRNA expression of FABP5 in PEO1 (n=4) and PEO4 (n=5) cells. P=0.012. **(i-j)** Relative mRNA expression of GLUT1 in SKOV3 and -cisR(n=8. P=0.0089) (i) and in OVCAR5 treated with cisplatin for 0, 6, 12 or 24 hours (n = 3) (j). n for mRNA expression measurement (g-j) represents biological replicates. **(k, m)** Representative bright field and SRS images of SKOV3 and SKOV3-cisR fed with PA-d31 (k) and OA-d34 (m) in various concentrations for 6 h. Scale bar: 20 µm. **(l, n)** Quantitation of SRS signal intensity for (k and m). n=38,33,21,37,35,35,42and23 technical replicates. P=2.2×10^{-7},0.0016 and 0.019. Data in bar charts (b and g-j,) are presented as means + SD. Data in dose-response curve (c and d) are presented as mean ± SD. For box plots (f, l and n), the outer box, inner box, lines, whiskers and circles indicates 25% to 75% of data, mean, medium, SD, maxima and minima respectively. All statistical significance was analyzed using one-sided Student’s t test. * $P < 0.05$, ** $P < 0.01$, and *** $P < 0.001$. Source data are provided in Source Data file.

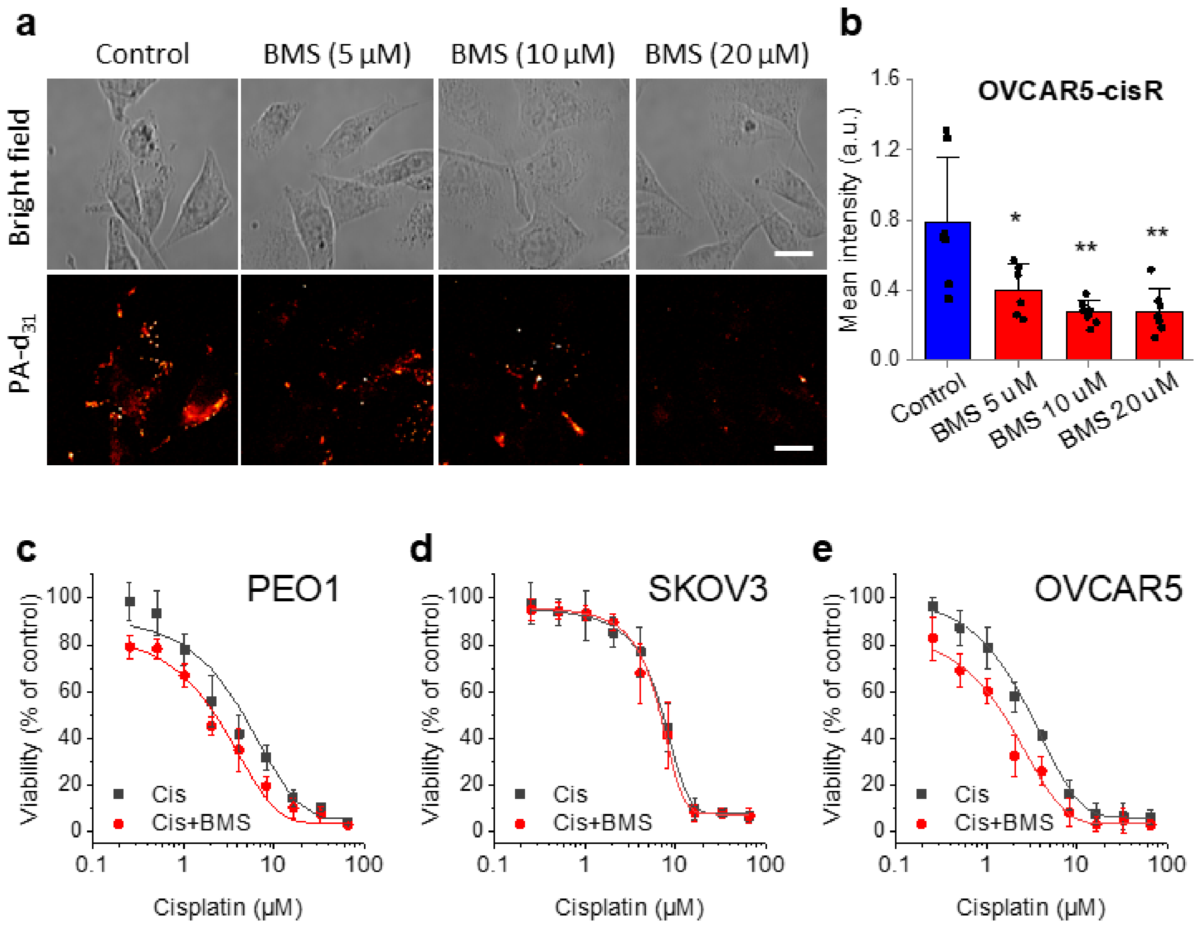

Supplementary Fig. 5. BMS inhibits FA uptake and sensitizes OC cell to cisplatin treatment. (a) Representative bright field and SRS images of OVCAR5-cisR cell after FA transporter inhibitor BMS309403 (BMS) treatment at 5 µM, 10 µM or 20 µM for 24 hours with the incubation of 100 µM PA-d31 for 6 hours. Scale bar: 20 µm. n=6, 5, 6 and 6 technical replicates. **(b)** Quantification of C-D SRS signal intensity from OVCAR5-cisR after treatment with BMS at 5 µM, 10 µM or 20 µM for 24 hours during concomitant incubation of 100 µM PA-d31 for 6 hours. Data are presented as means + SD; n=6, 5, 6 and 6 technical replicates; one-sided Student's t test; P=0.019, 0.0055 and 0.0056. * *P* < 0.05, ** P<0.01. **(c-e)** Dose-response to cisplatin with or without BMS treatment for PEO1 (c), SKOV3 (d) and OVCAR5 (e) cells. n = 3 biological replicates. The results are shown as means ± SD. Source data are provided in Source Data file. Source data are provided in Source Data file.

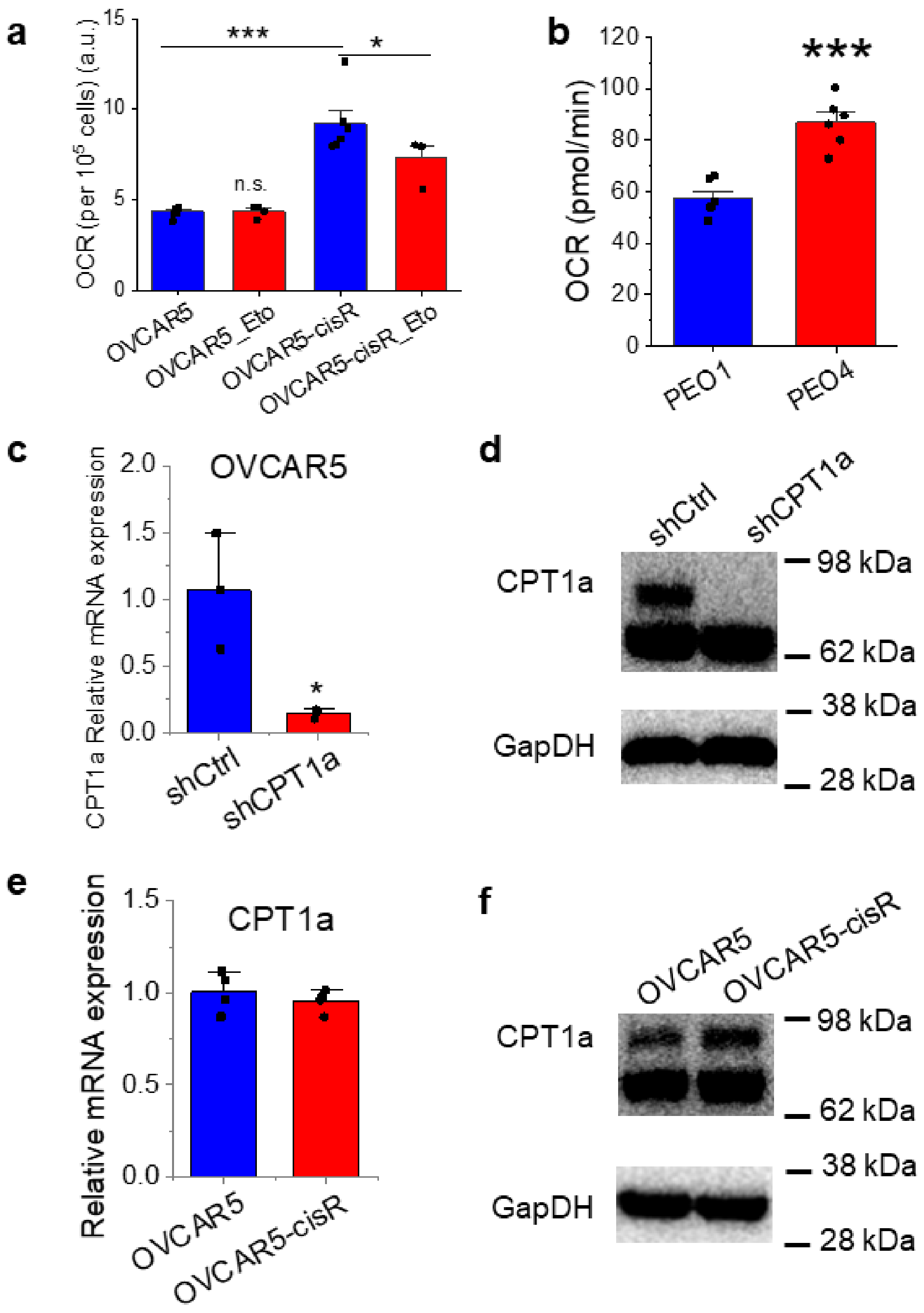

Supplementary Fig. 6. FA uptake contributes to cisplatin resistance by increasing FAO. (a) Quantification of OCR for OVCAR5 and OVCAR5-cisR cells treated with (n=4) or without (n=6) etomoxir (40 μM) measured by using the extracellular oxygen consumption kit (Abcam). P=0.00044 and 0.041.**(b)** Quantification of OCR for PEO1 and PEO4 cells measured through Seahorse XF Analyzers (Seahorse Bioscience). n = 6. P=8.9×10^{-5}. n for OCR measurement (a-b) represents technical replicates. **(c)** Relative mRNA expression levels of CPT1a in OVCAR5-cisR shCtrl and shCPT1a cell. n =3. P=0.033. **(d)** Western Blot of CPT1a and GAPDH for OVCAR5-cisR cells transduced with shCtrl and shRNA targeting CPT1a (shCPT1a cell). n=3**(e)** Relative mRNA expression levels for CPT1a in OVCAR5 and OVCAR5-cisR cells. n =4. **(f)**

Western Blot for CPT1a and GAPDH in OVCAR5 (n=2) and OVCAR5-cisR (n=3) cells. n for mRNA expression and western blot measurement (c-f) represents biological replicates. The results in all bar chart (a-c, e) are shown as means + SD. All statistical significance was analyzed using one-sided Student's t test; * $P<0.05$. *** $P<0.001$. n.s. $P>0.05$. Source data are provided in Source Data file.

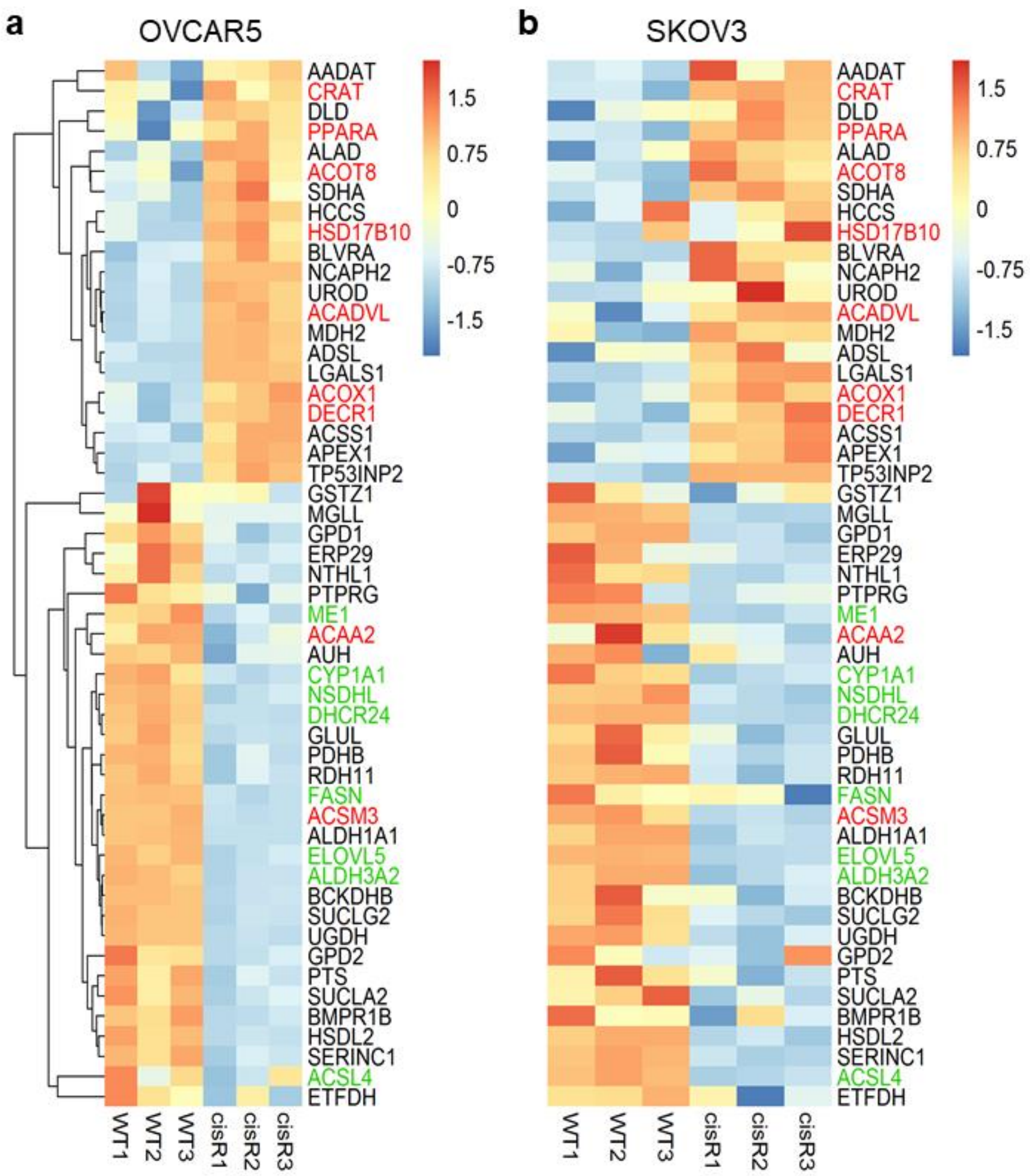

Supplementary Fig. 7. FA uptake and FAO are linked to cisplatin resistance Heatmap of lipid metabolism related genes, as analyzed by RNA-sequencing in OVCAR5 **(a)** and SKOV3 **(b)** cell line pair. FAO related genes are highlighted in red, and lipogenesis related genes are depicted in green.

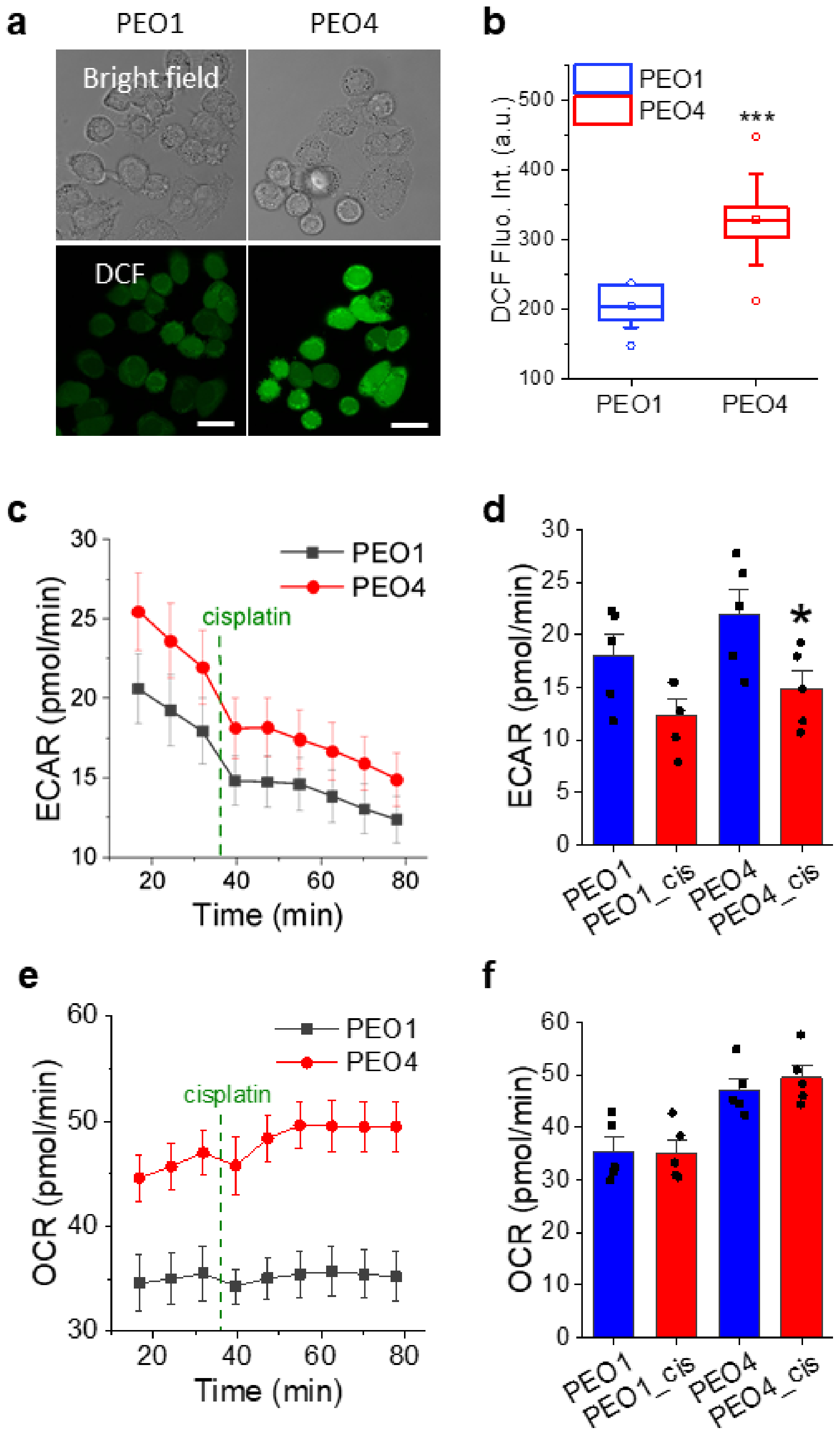

Supplementary Fig. 8. Increased FA uptake and oxidation supports cancer cell survival under cisplatin-induced oxidative stress. (a) Representative bright field and fluorescent images of PEO1 and PEO4 cells using the DCFDA cellular ROS assay kit. Scale bar: 30 µm. **(b)** Quantification of DCF fluorescent signal intensity for PEO1 and PEO4 cells. The bound of outer box indicates 25% to 75% of data; inner box indicates

mean; lines represent medium; whiskers indicate SD; circles indicate maxima and minima of data; n=10; P=0.00013. **(c)** ECAR profile of PEO1 and PEO4 cells after treatment with 13.2 μM cisplatin measured by Seahorse. P=0.041. **(d)** Quantification of PEO1 and PEO4 cells' ECAR before and 30 minutes after 13.2 μM cisplatin treatment. **(e)** OCR profiles of PEO1 and PEO4 after 13.2 μM cisplatin treatment measured by Seahorse. **(f)** Quantification of OCR for PEO1 and PEO4 cells before and 30 minutes after treatment with 13.2 μM cisplatin measured by Seahorse. The data in all Seahorse kinetic profiles (c and e) are shown as means ± SD. Data in all bar charts (d and f) are presented as means + SD. n = 5. All n represents technical replicates. All statistical significance was analyzed using two-sided Student's t test; * P<0.05. *** P<0.001. Source data are provided in Source Data file.

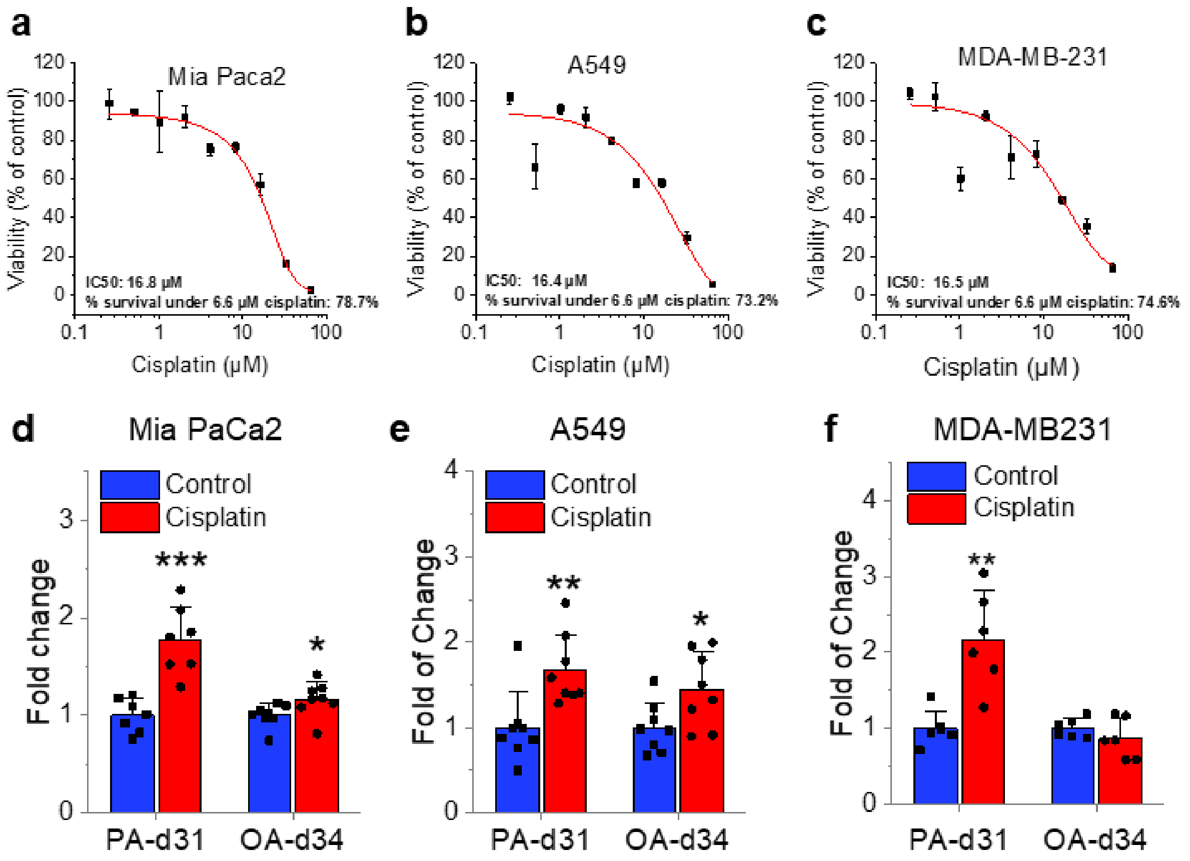

Supplementary Fig. 9. Cisplatin induced FA uptake is a universal metabolic feature in multiple types of cancers. (a-c) Dose-response to cisplatin for Mia Paca2 (a) A549 (b) and MD-MBA231 (c). The data are shown as means ± SD; n = 3. **(d-f)** Quantitation of C-D signal in Mia Paca2 (n=7 for PA-d31 and n=8 for OA-d34) (d), A549 (n=8) (e), and MDA-MB-231 (n=6) (f) cells treated with or without cisplatin by fold of change. Data in all dose-response curve (a-c) are presented as mean ± SD; Data in all bar charts (d-f) are presented as means + SD. all statistical significance was analyzed using one-sided Student's t test; P=0.00029, 0.026, 0.0031, 0.016 and 0.0024; All n represents technical replicates. * P<0.05. ** P<0.01. *** P<0.001. Source data are provided in Source Data file.

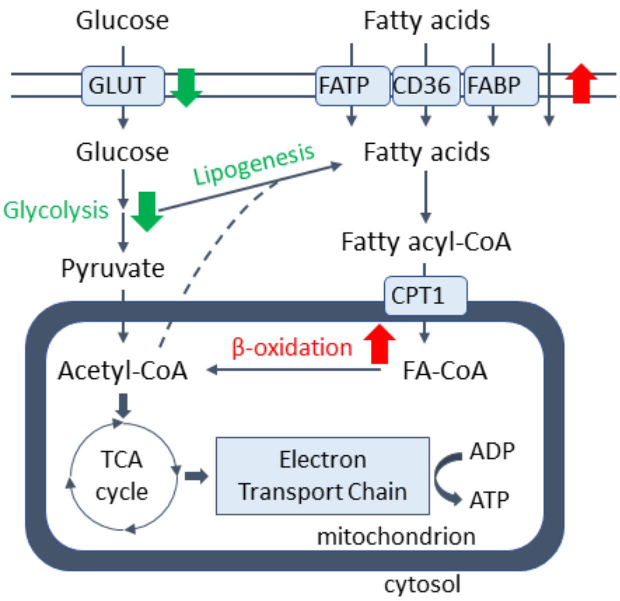

Supplementary Fig. 10. Diagram showing metabolic reprogramming in cisplatin-resistant ovarian cancer cells. Proposed mechanism illustrates the cellular metabolism switch from glycolysis to FAO in cisplatin-resistant ovarian cancer cell.

Supplementary Table 1. Summary of quantitation results of glucose-d_7, PA-d_{31}, and OA-d_{34} and IC_{50}s for cisplatin in 4 pairs of parental and cisplatin-resistant ovarian cancer cells.

Cell lines	IC_{50} for cisplatin (μM)	Intensity (area fraction (%))		
		G-d7	PA-d31	OA-d34
PE01	4.72	3.88	6.11	17.25
PE04	13.57	0.93	13.69	21.61
SKOV3	10.07	3.39	9.88	6.24
SKOV3-CisR	17.29	0.84	15.79	10.97
OVCAR5	8.26	3.64	10.37	18.35
OVCAR5-CisR	17.43	2.51	15.91	23.28
COV362	7.19	3.14	4.44	31.00
COV362-cisR	15.17	2.03	14.88	41.43

Quantitation of glucose-d7, PA-d31, and OA-d34 are shown as area fraction of C-D signal out of total cellular area. Only mean values are shown.

Supplementary Table 2: Patient characteristics for primary cells used for metabolic index calculation (n = 11 patients). Tumor specimens were obtained at the time of cytoreductive surgery either upfront (n = 4) or after neoadjuvant chemotherapy (n = 7). Platinum resistance was defined as disease recurring within 6 months from completing carboplatin based chemotherapy, as assessed clinically, by CA125 criteria or CT scans.

**Carboplatin Sensitive**		**Carboplatin Resistant**	
Patient ID	Chemotherapy before surgery	Patient ID	Chemotherapy before surgery
1	Yes	8	Yes
2	Yes	9	Yes
3	No	10	Yes
4	No	11	Yes
5	Yes		
6	No		
7	Yes		

Supplementary Table 3. Primer sequences used for RT-PCR measurement.

Gene name	Forward sequence	Backward sequence
CPT1a	TCCAGTTGGCTTATCGTGGTG	TCCAGAGTCCGATTGATTTTTGC
FABP5	TGAAGGAGCTAGGAGTGGGAA	TGCACCATCTGTAAAGTTGCAG
FABP (PM)	GGAAGGAAATAGCAACAGTGG	TCCTACACGCTCACCATATAAGC
FATP1	CTTCGATGGCTATGTCAGCGA	AGCACGTCACCTGAGAGGTAG
FATP2	ATGCGAGAAAAGTTGGTGCT	TTTCATCACGGACAGGTTCA
FATP3	ATACCTGGGAGCGTTTTGTG	CCGCTGTCCTGTGTAGTTGA
FATP4	CTTTTCCAGCCGCTTCCACA	TGGCTGGCAGGGAATGCA
FATP5	AGCTCCTGCGGTACTTGTGT	AAGGTCTCCCACACATCAGC
FATP6	GCTGGGCCTTATAAGCACACA	CAACCTCAGTGGTTGCGACA
CD36	GGCTGTGACCGGAACTGTG	AGGTCTCCAACTGGCATTAGAA
FABP4	ACTGGGCCAGGAATTTGACG	CTCGTGGAAGTGACGCCTT
PPIA	CCCACCGTGTTCTTCGACATT	GGACCCGTATGCTTTAGGATGA
