# Supplementary material for: Metabolic reprogramming from glycolysis to fatty acid uptake and beta-oxidation in platinum-resistant cancer cells
Source: Nat Commun. 2022 Aug 5;13:4554. doi: 10.1038/s41467-022-32101-w (PMC9356138; doi:10.1038/s41467-022-32101-w)
Supplement: Supplementary file 3 — Description of Additional Supplementary Files [file 41467_2022_32101_MOESM3_ESM.pdf]

Description of Additional Supplementary Files

File Name: Supplementary Data 1
Description: RNA-sequencing analysis of lipid metabolism related genes in OVCAR5 and SKOV3 cell line pairs (resistant vs. sensitive). Genes from RNA-seq were normalized by trimmed mean of M-values (TMM) approach in edgeR package. Statistical test was done using quantileadjusted conditional maximum likelihood (qCML) method. P values for Multiple hypothesis testing were adjusted by Benjamini and Hochberg method (FDR).
