# Supplementary material for: Metabolic reprogramming from glycolysis to fatty acid uptake and beta-oxidation in platinum-resistant cancer cells
Source: Nat Commun. 2022 Aug 5;13:4554. doi: 10.1038/s41467-022-32101-w (PMC9356138; doi:10.1038/s41467-022-32101-w)
Supplement: Supplementary file 5 — Reporting Summary [file 41467_2022_32101_MOESM5_ESM.pdf]

nature portfolio

Corresponding author(s): Ji-Xin Cheng, Daniela Matei, Junjie Li

Last updated by author(s): May 16, 2022

Reporting Summary

Nature Portfolio wishes to improve the reproducibility of the work that we publish. This form provides structure for consistency and transparency in reporting. For further information on Nature Portfolio policies, see our Editorial Policies and the Editorial Policy Checklist.

Statistics

For all statistical analyses, confirm that the following items are present in the figure legend, table legend, main text, or Methods section.

n/a	Confirmed	
☐	☒	The exact sample size (*n*) for each experimental group/condition, given as a discrete number and unit of measurement
☒	☐	A statement on whether measurements were taken from distinct samples or whether the same sample was measured repeatedly
☐	☒	The statistical test(s) used AND whether they are one- or two-sided<br>*Only common tests should be described solely by name; describe more complex techniques in the Methods section.*
☒	☐	A description of all covariates tested
☒	☐	A description of any assumptions or corrections, such as tests of normality and adjustment for multiple comparisons
☐	☒	A full description of the statistical parameters including central tendency (e.g. means) or other basic estimates (e.g. regression coefficient) AND variation (e.g. standard deviation) or associated estimates of uncertainty (e.g. confidence intervals)
☐	☒	For null hypothesis testing, the test statistic (e.g. *F*, *t*, *r*) with confidence intervals, effect sizes, degrees of freedom and *P* value noted<br>*Give P values as exact values whenever suitable.*
☒	☐	For Bayesian analysis, information on the choice of priors and Markov chain Monte Carlo settings
☒	☐	For hierarchical and complex designs, identification of the appropriate level for tests and full reporting of outcomes
☒	☐	Estimates of effect sizes (e.g. Cohen's *d*, Pearson's *r*), indicating how they were calculated

Our web collection on statistics for biologists contains articles on many of the points above.

Software and code

Policy information about availability of computer code

Data collection	LabView
Data analysis	ImageJ, CellProfiler, MATLAB, R package edgeR, Microsoft Excel, Origin

For manuscripts utilizing custom algorithms or software that are central to the research but not yet described in published literature, software must be made available to editors and reviewers. We strongly encourage code deposition in a community repository (e.g. GitHub). See the Nature Portfolio guidelines for submitting code & software for further information.

Data

Policy information about availability of data

All manuscripts must include a data availability statement. This statement should provide the following information, where applicable:

- Accession codes, unique identifiers, or web links for publicly available datasets
- A description of any restrictions on data availability
- For clinical datasets or third party data, please ensure that the statement adheres to our policy

The RNA-seq data reported in this paper was accessible through Gene Expression Omnibus with accession ID: GSE148003. The remaining data are available in the article and supplementary information within this paper.

Field-specific reporting

Please select the one below that is the best fit for your research. If you are not sure, read the appropriate sections before making your selection.

☒ Life sciences ☐ Behavioural & social sciences ☐ Ecological, evolutionary & environmental sciences

For a reference copy of the document with all sections, see nature.com/documents/nr-reporting-summary-flat.pdf

Life sciences study design

All studies must disclose on these points even when the disclosure is negative.

Sample size	Sample sizes were predetermined based on the basis of prior experience, published standards in the field and standard deviation. The sample size for individual experiment was determined based on sample availability and the balance between experiment time and data standard deviation. Sample sizes are reported for each experiment in the manuscript.
Data exclusions	No data were excluded from analysis.
Replication	All attempts to replication were successful. Sample size for each experiment are indicated in the figure legends.
Randomization	All in vitro experiments were randomized. For animal study, mice in each group were randomly assigned to treatment groups.
Blinding	Animal study were blinded during data collection and analysis. In vitro study were keep blinded as possible but blinding for imaging experiment is not applicable due to cell's morphology feature. However, data collection was randomized to avoid potential operator bias.

Reporting for specific materials, systems and methods

We require information from authors about some types of materials, experimental systems and methods used in many studies. Here, indicate whether each material, system or method listed is relevant to your study. If you are not sure if a list item applies to your research, read the appropriate section before selecting a response.

Materials & experimental systems

n/a	Involved in the study	
☐	☒	Antibodies
☐	☒	Eukaryotic cell lines
☒	☐	Palaeontology and archaeology
☐	☒	Animals and other organisms
☐	☒	Human research participants
☒	☐	Clinical data
☒	☐	Dual use research of concern

Methods

n/a	Involved in the study	
☒	☐	ChIP-seq
☒	☐	Flow cytometry
☒	☐	MRI-based neuroimaging

Antibodies

Antibodies used	CPT1A Polyclonal Antibody, Proteintech Cat#15184-1-AP; RRID: AB_2084676 (1:1000)<br>GAPDH Monoclonal Antibody, Proteintech Cat#60004-1-Ig; RRID: AB_2107436; Clone#: 1E6D9 (1:2000)<br>Goat anti-mouse IgG (H+L), HRP conjugate Proteintech Cat#SA00001-1 RRID: AB_2722565 (1:10000)
Validation	CPT1A Polyclonal Antibody, Proteintech Cat#15184-1-AP validated in WB (https://www.ptgcn.com/products/CPT1A-Antibody-15184-1-AP.htm)<br>GAPDH Monoclonal Antibody, Proteintech Cat#60004-1-Ig validated in WB (https://www.ptgcn.com/products/GAPDH-Antibody-60004-1-Ig.htm)<br>Goat anti-mouse IgG (H+L), HRP conjugate Proteintech Cat#SA00001-1 validated in WB (https://www.ptglab.com/Products/Goat-anti-mouse-IgG-(H-L),-HRP-conjugate-secondary-antibody.htm)

Eukaryotic cell lines

Policy information about cell lines

Cell line source(s)	SKOV3, Mia Paca2, MDA-MB-231 and A549 cells were purchased from the American Type Culture Collection (ATCC) and PEO1 and PEO4 were from Sigma Aldrich. OVCAR5 cells were a generous gift from Dr. Marcus Peter, Northwestern University originally from Developmental Therapeutics Program (DTP) at the National Cancer Institute, and COV362 cells were from Dr. Kenneth Nephew, Indiana University originally obtained from Sigma Aldrich. The resistant cell lines SKOV3-cisR, COV362-cisR and OVCAR5-cisR were generated by treatment with 3 or 4 repeated or increasing doses of cisplatin for 24 hours. Surviving cells were allowed to recover for 3 to 4 weeks before receiving the next treatment.

March 2021

Authentication	Authentication of SKOV3, Mia Paca2, MDA-MB-231 and A549 was performed by ATCC through STR profiling. Authentication of COV362, PEO1 and PEO4 were performed by Sigma Aldrich through STR profiling. OVCAR5 was authenticated via Applied Biosystems AmpFISTR Identifiler testing with PCR amplification by DTP. Developed resistant cell were not further authenticated.
Mycoplasma contamination	All cell lines were tested to be mycoplasma negative.
Commonly misidentified lines (See ICLAC register)	There is no commonly misidentified cell lines in this study.

Animals and other organisms

Policy information about studies involving animals; ARRIVE guidelines recommended for reporting animal research

Laboratory animals	7–8-week-old female NSG mice (Jackson Labs Cat#JAX:00555) and 6-8 weeks old female athymic nude mice (Foxn1nu, Envigo) were used in this study.
Wild animals	There is no wild animals involved in this study.
Field-collected samples	There is no field-collected samples involved in this study.
Ethics oversight	Animal studies were approved by the Institutional Animal Care and Use Committee (IACUC) at Northwestern University .

Note that full information on the approval of the study protocol must also be provided in the manuscript.

Human research participants

Policy information about studies involving human research participants

Population characteristics	De-identified high grade serous ovarian tumors (HGSOC) and malignant ascites fluid specimens from OC patients
Recruitment	Samples were obtained at the time of cytoreductive surgery either upfront or after neoadjuvant chemotherapy (interval debulking surgery) at the Northwestern University School of Medicine
Ethics oversight	Northwestern University School of Medicine under an IRB approved protocol (STU00202468)

Note that full information on the approval of the study protocol must also be provided in the manuscript.
